# Rationale and design for cognitive behavioral therapy for anxiety disorders in children with autism spectrum disorder: a study protocol of a randomized controlled trial

**DOI:** 10.1186/s13063-018-2591-x

**Published:** 2018-04-02

**Authors:** Tina R. Kilburn, Merete Juul Sørensen, Mikael Thastum, Ronald M. Rapee, Charlotte Ulrikka Rask, Kristian Bech Arendt, Per Hove Thomsen

**Affiliations:** 1 Research Unit, Centre of Child and Adolescent Psychiatry, Aarhus University Hospital, Risskov, Denmark; 2 Unit C for School-aged Children, Centre of Child and Adolescent Psychiatry, Aarhus University Hospital, Risskov, Denmark; 3 Department of Psychology and Behavioural Science, Aarhus University, Aarhus, Denmark; 4 Centre for Emotional Health, Department of Psychology, Macquarie University, Sydney, Australia; 5 Research Unit & Unit C for School aged Children, Centre of Child and Adolescent Psychiatry, Aarhus University Hospital, Risskov, Denmark; 6 Centre of Child and Adolescent Psychiatry, Aarhus University Hospital, Risskov, Denmark

**Keywords:** Autism spectrum disorder, anxiety disorders, children, cognitive behavioral therapy

## Abstract

**Background:**

Autism spectrum disorder (ASD) is found in approximately 1% of the population and includes core symptoms that affect general and social development. Beside these core symptoms, it is suggested that up to 60% of children with ASD suffer from comorbid anxiety disorders which may further affect educational, social and general development as well as quality of life. The main goal of this study is to examine the effectiveness of a manualized cognitive behavioral therapy (CBT) anxiety program adapted for children with ASD.

**Methods:**

This study is a randomized controlled trial (RCT). Fifty children with ASD and anxiety, aged 7 to 13 years, will be randomly assigned to group CBT or a wait-list control (WL) condition. The design will follow a two (CBT and WL) by two (pre–post assessment) mixed between–within design. The control group will receive intervention after the waitlist period of 13 weeks. Primary outcomes are diagnostic status and severity of the anxiety disorders, measured with The Anxiety Disorder Interview Schedule for DSM-IV, Parent and Child Versions. Secondary outcomes are parent and child ratings on questionnaires on the child’s level of anxiety and impact on everyday life. Additional outcomes entail information gathered from parents, child and teachers on the child’s behavior and negative self-statements, together with social and adaptive skills. Follow-up data will be collected 3 months after intervention.

**Discussion:**

This study aims to evaluate the effectiveness of a manualized CBT program in Danish children with ASD and anxiety within a mental health clinic setting. The hypothesis is that training anxiety reduction skills will decrease anxiety in children, as well as ensure better psychosocial development for the child in general.

**Trial registration:**

https://ClinicalTrials.gov (NCT02908321). Registered 19th of September 2016.

**Electronic supplementary material:**

The online version of this article (10.1186/s13063-018-2591-x) contains supplementary material, which is available to authorized users.

## Background

Autism spectrum disorders (ASD) include deviations and delays in the development of social interaction and communication patterns, restricted stereotyped and repetitive behaviors, as well as, often, specific areas of interests [1].

ASD occur in children and adolescents with a prevalence of approximately 60–115 per 10,000, with numbers increasing in recent years [2–6]. Approximately half of children diagnosed with ASD have a relatively poor psychosocial development into young adulthood, with difficulties concerning work, friendship, and independence, leaving them socially isolated and reliant on social, community, and family care [7].

Individuals with ASD have a high risk of developing co-morbid disorders [8–10]. According to Gillberg and Coleman [8, 9], the co-morbid disorders in ASD seem to have an even greater impact on disability, suffering and behavior than the ASD itself. Attention Deficit Hyperactivity Disorder (ADHD) [11–15], anxiety disorders [11–13, 15–18], oppositional defiant disorder [12, 15, 16], and obsessive compulsive disorders (OCD) [11, 12, 17] are the most prominent co-morbid disorders in children with ASD. Therefore, treatment of co-morbid disorders may potentially add to improvements in quality of life and general functioning, even though ASD itself is not ‘curable’ [10].

Several studies show that certain types of anxiety disorders, especially specific phobias and separation anxiety, are far more common among children with ASD than previously assumed [11, 12, 18]. While anxiety disorders are seen in 3–5% of children [19], among children with ASD, comorbid anxiety disorders have been reported in 40–60% [11, 20, 21].

Cognitive behavioral therapy (CBT) has been shown to be effective in the treatment of anxiety disorders in typically developing children [22, 23]. It is, however, still not evident whether children suffering from ASD and comorbid anxiety disorders will respond to standard CBT for anxiety disorders [24] and, thus, clinical judgement has typically assumed that the response will be poor due to the core difficulties associated with ASD. As a result, treatment of anxiety disorders in children with ASD has mostly been based on general principles for behavioral adaptation and stress reduction, including environmental structuring, visual guiding, social skills training, and pharmacological treatment with selective serotonin reuptake inhibitors. However, in recent years, research has begun to evaluate the adaptation of CBT for anxiety disorders in children and adolescents with ASD [25]. The few existing studies suggest that CBT programs specifically designed for children with ASD and anxiety disorders are highly efficient in treating the latter [26, 27] and, thereby, in reducing the comorbid symptoms that otherwise untreated may lead to additional impairment in daily life functioning.

Meta-analytic reviews show CBT to be efficient in reducing anxiety symptoms in children and adolescents with ASD, with overall effect sizes for clinician- and parent-rated outcomes measures of anxiety disorders across all of the included studies yielding Cohen’s *d* values of 1.19 and 1.21, respectively [26]. Although the effects are looking promising, the evidence is still limited and, more importantly, no studies have been conducted in real-world, outpatient mental healthcare settings.

Further, although ASD itself is incurable, some symptoms might be exaggerated by comorbid anxiety disorders and may improve if these are successfully treated. Only a few very small studies have looked at the effects of CBT on ASD symptoms, which might in turn improve children’s quality of life [28, 29].

The objective of this study is to investigate the effect of a manualized CBT program for anxiety disorders adapted for children with ASD within a mental health clinic setting using a wait-list controlled design. The primary outcome investigated will be treatment effects on anxiety disorder diagnoses and the severity of the disorder. Further, we will investigate secondary outcomes related to the anxiety disorder’s impact on the child’s everyday life together with additional outcomes investigating the child’s general functioning, co-morbid psychiatric disorders (OCD, ADHD and depression), and level of ASD symptoms (social and communicative skills).

## Method

### Study design

This study examines the effectiveness of a manualized CBT program using a randomized controlled trial (RCT) design allocating children to either treatment or a wait-list (WL) control condition.

The two groups are then followed prospectively to assess the effectiveness of the CBT program. See Additional file 1 for the completed SPIRIT (Standard Protocol Items: Recommendation for Interventional Trials) checklist of recommended items to address in a clinical trial process.

### Procedure

#### Participants

Children aged 7–13 years, referred to the Centre of Child and Adolescent Psychiatry, Aarhus University Hospital, Denmark, diagnosed with ASD, and experiencing anxiety symptoms impairing their quality of life can be referred to the study by their treating clinician.

Children and parents who are not able to participate in a standard child psychiatric assessment due to inability to speak Danish or who are unable to attend assessment due to, for example, parental mental disorder, inability to leave home, etc., will be excluded prior to randomization. These children will receive individual specialized assessment and treatment arranged by the mental health clinic.

**Fig. 1:**
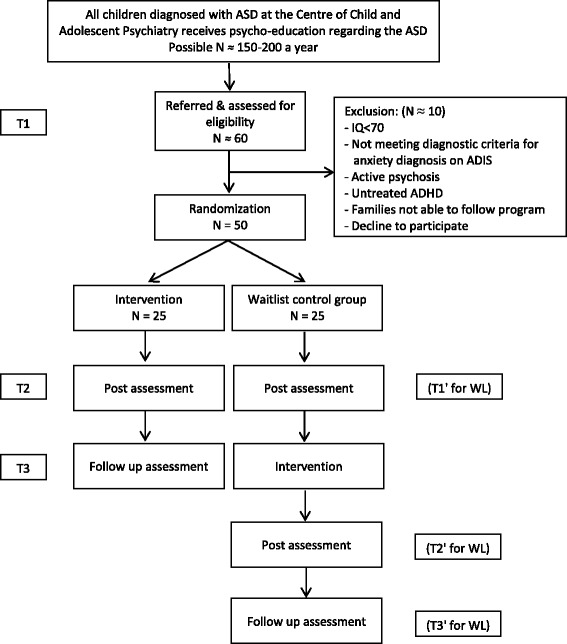
Different stages of the study procedure

Additional exclusion criteria are child mental retardation (IQ < 70), active psychosis, untreated ADHD, families who have not received psycho-educational intervention for ASD, or families considered not able to follow the CBT program (e.g., due to history of recurrent non-attendance, severe interfering family problems rendering the parents unable to participate in sessions (i.e., grave illness or death in the immediate family, parents in conflict over custody, etc.)). This exclusion will occur before randomization, and thus will not affect the trial’s internal validity (Fig. 1).

During the initial enrolment process, and before consenting to participation, the study participants will be advised to carefully consider the required investment of time and effort involved in the treatment. This will be undertaken to minimize any negative impact associated with loss to follow-up, and consequently likely withdrawal can be made prior to randomization [30].

Based on the clinical judgement of the child psychiatrist responsible for the clinical treatment of the child, non-responders (i.e. no or limited decrease in anxiety symptoms at follow-up) will be offered either additional treatment, which may include individual CBT (if deemed appropriate) and/or medication, or no further treatment.

Based on numbers from previous years retrieved from the electronic patient’s journal, approximately 150–200 children are diagnosed with ASD at the Centres of Child and Adolescent Psychiatry, Aarhus University Hospital, each year. According to the literature, it can be expected that slightly less than half of these children will have an additional anxiety disorder [11], leaving a sufficient number of children to be approached for recruitment.

#### Study procedure

Children referred to this study will have been diagnosed with ASD according to the International Classification of Diseases, 10th edition (ICD 10) [31] at a prior date at the Centre of Child and Adolescent Psychiatry, Aarhus University Hospital, Denmark. The assessment was performed by an interdisciplinary team consisting of experienced psychologists, psychiatric nurses, educationalists, and child psychiatrists and included (1) a structured life history, including the child’s medical history (parents), Autism Diagnostic Observation Schedule (ADOS) assessment (child) (where deemed appropriate) [32], structured observation in either school or in the clinical setting, medical examination (child), assessment of cognitive ability with the Wechsler Intelligence Scale for Children–Fourth edition (WISC-IV) [33] or Reynolds Intellectual Assessment Scales (RIAS) [34] (child).

Before entering the program, it is important that families have a basic understanding of how to support the core difficulties in children with ASD, since this may reduce some of the experienced anxiety in itself and, as such, is not a part of the CBT intervention. Thus, following assignment of an ASD diagnosis, all families have been offered a brief psycho-educational intervention consisting of group-based, autism-focused parent counselling, comprising four, 2-hour group sessions regarding behavioral management, structuring, visual aids, and stress reduction, as well as up to three individual counselling sessions focusing on the specific problems of the individual child and family. Children with additional clinically relevant anxiety symptoms as assessed by the interdisciplinary team are then invited to participate in an assessment for the current study.

Children who have been previously diagnosed with ASD and now re-referred to the clinic with a possible anxiety disorder will be directly invited to participate in the study if they and their parents had previously received individual ASD counselling when the diagnosis was originally given.

After being referred to assessment for the study and before randomization, possible anxiety disorders are assessed by using the structured interview Anxiety Disorders Interview Schedule for DSM-IV: Parent & Child interview schedule (ADIS-IV-C/P) [35], which will be described in detail in the outcome section below. The ADIS-IV-C/P and questionnaires regarding secondary and other outcomes are distributed approximately 2–3 weeks before commencement of either CBT treatment or the WL period.

Treatment then runs for 10 sessions over a 13-week period. At the end of treatment, for ethical purposes, the WL control group will be offered the same CBT program as the trial group. This will be carried out in groups with fewer than five children if some of the WL children decide not to accept this offer.

Following treatment or WL period, and again at a 3 month follow-up (after treatment), the families will be interviewed with the ADIS-IV-C/P and asked to answer the questionnaires again.

### Randomization

To control for the potential experience of therapists and to limit time between assessment and treatment, allocation to CBT treatment or WL control group will be performed using blocked randomization. Each block will comprise 10 participants and, within each block, allocation to either the WL control group or the treatment group will in a 1:1 allocation ratio. For each child, at least one of their parents participates and the total number of persons in each group may therefore vary.

In practice, randomization will be performed as follows:

From the total pool of 252 possible different sequences of five CBT and five WL allocations (e.g. {WL, WL, WL, CBT, CBT, WL, CBT, CBT, WL, CBT}), one is picked at random for each of five blocks and the allocations are written on a text file together with a unique five-digit subject identification number. These block-specific files are being generated by a statistical consultant (blind for the therapists and the project manager) and each file will not be opened before the next (the first) 10 children are ready for randomization. The children are numbered 1 to 10 before opening the file and child 1 will then be allocated to the condition noted for subject 1 in the file, child 2 to the condition noted for subject 2, etc.

### Intervention

The group-based manualized CBT intervention consists of The Cool Kids Anxiety Program: Autism Spectrum Disorder Adaptation (Cool Kids ASD), 2nd Edition [36] (edited and enhanced from 1st edition) [37]. The program is a modification of the Cool Kids Child and Adolescent Anxiety Program [38], which has been found to be efficacious in a Danish context with typically developing children with anxiety [39, 40].

The first modification of the original program involved extending the program in duration (up to 6 months) and in number of sessions (from 10 to 12) including the use of more visual aids and structured worksheets. The adaptations to the Cool Kids program were made in order to account for the visual and concrete learning style of children with high-functioning autism [41], covering the recognition of anxious feelings and somatic reactions to anxiety, simplified cognitive restructuring exercises, coping self-talk, exposure to feared stimuli, and relapse prevention. Moreover, the largest components of the program were devoted to relaxation and exposure since they involve more concrete exercises and place less emphasis on the children’s communication skills, which are often markedly impaired. In addition, a cognitive therapy component was still included in the program, but the information in the cognitive activities was simplified, as were the tasks involving generating helpful and unhelpful thoughts and thought challenging. Rather than relying on the children’s impaired communication skills when generating their own helpful and unhelpful thoughts, the children identified helpful and unhelpful thoughts from pre-prepared worksheets including lists of possible alternatives. A parent-based group CBT manual was also adapted from the Cool Kids program to use with the families in concurrent sessions to the child program [25].

Based on common experiences with the Cool Kids ASD program between the original developer and the Danish Centre of Child and Adolescent Psychiatry, Aarhus University Hospital, it was decided to make a second version of the program. However, most of the content remains the same, the duration and numbers of sessions have been shortened and several explanatory components have been removed, leaving more emphasis on the coping methods and exposure [36].

The second version of the program used in this study is conducted in 10 sessions over approximately 13 weeks. The group sessions are conducted weekly, with a 1 week break after sessions 3, 6, and 8 to allow participants time to implement strategies in daily life. Each session lasts 2 hours and includes time spent working with the children alone, time with the parents alone, and time with parents and children together. See Table 1 for content overview of each session.

**Table 1:** Session content

Session number	Content overview for children	Content overview for parents
1.	• What are feelings • What is anxiety • Introduction to hand puppets Calvin & Austin • List of worries • Anxiety and my body • Rewards menu	• Nature of child’s anxiety, its development and its connection to ASD • Aim of program • Setting goals • Principles of using rewards effectively to reinforce child behavior
2.	• Cool breathing • Relaxation • Feel-good activities • The worry scale	• Introduction and principles of the three relaxation methods • The importance of practice • Introduction and principles behind cognitive restructuring
3.	• Helpful and unhelpful thoughts ▪ How to identify them ▪ A prepared list of helpful thoughts ▪ How to use helpful thoughts	• Realistic, helpful thinking for children with ASD • Avoidance and anxiety • Introduction to exposure • Principals and application of exposure hierarchies (stepladders)
4.	• Fighting fear by facing fear • Stepladders	• Stepladders
5.	• Review of stepladder progress • A small step first	• Parent’s anxiety traps • Alternative parental strategies, including encouraging greater child independence • Introduction and principles behind ‘A small step first’
6.	• Review of stepladder progress • Review of relaxation skills • Create a new stepladder	• Challenges to exposure • Overcoming barriers to stepladder practice • Planning in session exposure
7.	• Review of stepladder progress • Review of helpful thinking • In-session exposure	• Social skills and assertiveness • How to train social skills • In-session exposure
8.	• Review of stepladder progress • Review of a small step first • In-session exposure	• Creating creative stepladders • Reviewing alternative parental strategies • In-session exposure
9.	• Review of stepladder progress • How can I help others	• Structured problem solving • Reviewing goals
10.	• Review of stepladder progress • Setbacks and how to deal with them • What have Cool Kids ASD changed • Congratulation and reception	• Maintenance and setback • Reviewing goals and planning new ones • Planning strategies to use for future challenges of high-risk times such as changing school

Each CBT group consists of five children with one or both parents attending as well as a main group therapist and a co-therapist. The two main group therapists are psychologists with extensive psychiatric and CBT experience and the three co-therapists are experienced psychiatric nurses or educationalists with knowledge of ASD and anxiety. All have had specialized training in the Cool Kids ASD, 2nd edition program [36] by one of the original developers of the program. The aim is for the two main therapists to have five groups each with the three co-therapists distributed as equally as possible between groups.

#### Treatment fidelity

All sessions will be videotaped and adherence to the manual will be assessed by a research assistant trained in the Cool Kids ASD program through observation of a random selection of 2 out of the complete 10 sessions for each running program using a content checklist.

#### WL controls

During the 13 weeks of waiting, the families are advised to contact their allocated treating clinician if issues arise that need to be dealt with regarding the child’s mental health. After post-WL assessment, the families are offered the opportunity to take part in the Cool Kids ASD treatment program as a separate group (Fig. 1).

#### Adherence and drop outs

Treatment adherence is assessed by recording the number of completed CBT sessions. When applicable, participants are asked for their reasons for poor adherence or dropout. In the case of dropout, data collection is sought to continue as planned with as many outcome measures as possible.

### Outcome measures (Table 2)

#### Primary outcome

The primary outcome measure will be diagnostic status of the primary anxiety disorder measured with ADIS-IV-C/P [35]. ADIS-IV-C/P is a structured interview conducted with both child and parents designed to assess for current episodes of anxiety disorders, and to permit differential diagnosis among the anxiety disorders according to DSM-IV criteria. Further to the assessment of anxiety disorders, the ADIS-IV-C/P allows for assessment of other disorders such as depression, dysthymia, oppositional disorder, conduct disorder, and ADHD. In this study, only the sections regarding anxiety disorders along with OCD, depression, and dysthymia will be assessed.

**Table 2:** Overview of measurements

		CBT			WL			
Outcome	Scale	T1	T2	T3	T1	T1’	T2’	T3’
Sociodemographics	Developed by authors	✓			✓			
Child anxiety disorder	ADIS-IV-C/P	✓	✓	✓	✓	✓	✓	✓
Child co-morbid disorder (obsessive compulsive disorder and depression)	ADIS-IV-C/P	✓	✓	✓	✓	✓	✓	✓
Child anxiety symptoms	SCAS	✓	✓	✓	✓	✓	✓	✓
Life interference from anxiety	CALIS	✓	✓	✓	✓	✓	✓	✓
Attention deficit hyperactive disorder	ADHD-RS	✓	✓	✓	✓	✓	✓	✓
Social and communicative skills	SRS	✓	✓	✓	✓	✓	✓	✓
Behavioral and emotional problems	SDQ	✓	✓	✓	✓	✓	✓	✓
General measure of negative self-statements	CATS	✓	✓	✓	✓	✓	✓	✓
Child adaptive skills	ABAS-II	✓		✓		✓		✓
Parent quality of life	WHO-5 + PSE	✓	✓	✓	✓	✓	✓	✓
Program evaluation	ESQ		✓				✓	

*T1* baseline assessment, *T2 + T1’* 13 weeks after baseline, *T3* 25 weeks after baseline, *T2’* 13 weeks after T1’-baseline, *T3’* 25 weeks after T2’-baseline. Primary outcome: *ADIS-IV-C/P* Anxiety Disorders Interview Schedule for DSM-IV: Parent & Child interview schedule; Secondary outcomes: *SCAS* Spence Children’s Anxiety Scale, *CALIS* Children’s Anxiety Life Inference Scale; Other outcomes: *ADHD-RS* Attention Deficit/Hyperactive Disorder-Rating Scale, *SRS* Social Responsiveness Scale, *SDQ* Strengths and Difficulties Questionnaire, *CATS* Children’s Automatic Thoughts Scale, *ABAS-II* Adaptive Behavior Assessment System, Second Edition, *WHO-5* WHO-five Well-being Index + 10 questions regarding depression from Present State Examination, *ESQ* Experience of Service Questionnaire

The disorders are rated with a clinical severity rating (CSR) from 0 (no interference) to 8 (extreme interference) with severity ratings of 4 or above signifying the presence of a clinical disorder. The assessor’s CSR score, which is based on a combination of the parent and child’s CSR score plus a clinical evaluation of the extent of severity, will be used in assessing the disorder with the most impairing diagnosis considered the primary diagnosis.

The ADIS-IV-C/P has previously demonstrated good-to-excellent 7–14 days test–retest reliability for the presence of specific diagnoses (Cohen’s Kappa [κ] range for different diagnoses = 0.71–0.84 for children interviews and 0.73–0.92 for parent interviews) [42]. High concurrent validity against the Multidimensional Anxiety Scale for Children [43] has also been demonstrated [44].

The ADIS-IV-C/P assessment will be performed by a group of trained psychiatrists, psychologists or psychology students blinded to treatment conditions under supervision of a senior assessor at the Anxiety Disorder Clinic for Children and Adolescents, Department of Psychology and Behavioural Science, Aarhus University.

Inter-rater reliability will be conducted by two trained assessors based on 10% of the video recorded interviews selected randomly.

**Fig. 2:**
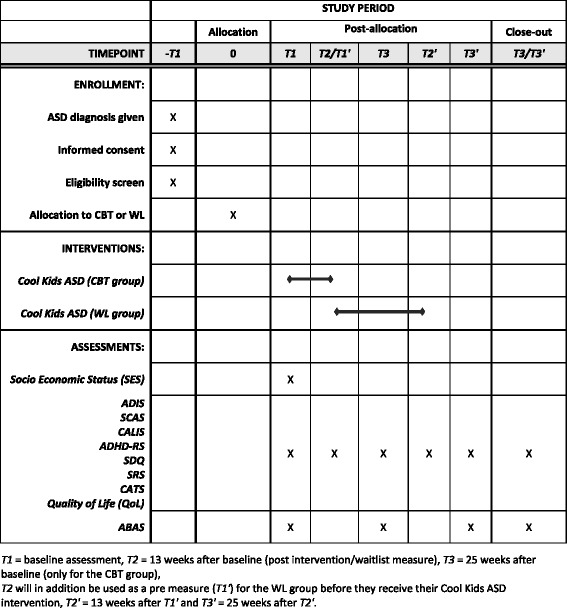
Schedule of enrollment, interventions, and assessments. Completed SPIRIT 2013 figure of recommended content for the schedule of enrolment, interventions, and assessments

For the CBT group, the ADIS-IV-C/P interview will be completed at pre, post, and follow-up 3 months after the last session (T1, T2, and T3) and pre and post for the WL group (T1 and T2). A similar set of measures at the post and 3-month follow-ups will be obtained from the WL group after their receipt of the CBT treatment following the WL period (T1’ = T2, T2’, and T3’). For an overview of the schedule of enrolment, interventions, and assessments please see Fig. 2 for the completed SPIRIT Figure.

#### Secondary outcomes

Secondary outcome measures are Spence Children’s Anxiety Scale (SCAS) [45] and Children’s Anxiety Life Inference Scale (CALIS) [46].

SCAS [45] is a questionnaire for children and parents assessing the severity of anxiety symptoms broadly in line with the dimensions of anxiety disorders proposed by the DSM-IV. It consists of 44 items for the children and 39 for the parents rated on a Likert scale ranging from 0 (never) to 3 (always). The scale assesses six domains of anxiety disorders, including generalized anxiety, panic/agoraphobia, social phobia, separation anxiety, OCD, and physical injury fears (specific phobia). These scales can be scored separately or added together for an overall anxiety disorder score. The Danish version of SCAS has shown excellent internal consistency for the total scale (α = 0.89) in a sample of youths with anxiety disorders and good test–retest reliability after 2 weeks (*r* = 0.84) and 3 months (*r* = 0.83) [47].

CALIS [46] is designed to assess life interference attributed to fears and worries from the child and parent perspectives. The measure targets interference in the child’s life (e.g., school, leisure time, and friendships) rated on nine items and in the parent’s/family’s life (e.g., relationships with friends and family, career, and level of stress) rated on 16 items. The items are rated on a Likert scale with the interference rated from 0 (not at all) to 4 (a great deal) and the scale has demonstrated satisfactory internal consistency on subscales for both youth (α range = 0.70–0.84) and parent ratings (α range = 0.75–0.90) and moderate stability for a 2-month retest period (*r* range = 0.62–0.91) [46].

#### Other outcomes

ADHD will be assessed using the Attention Deficit/Hyperactive Disorder-Rating Scale (ADHD-RS) [48], and depression and OCD with the parts of the ADIS that address these disorders [35].

Further, social and communicative skills will be assessed using parent and teacher rating scales from the Social Responsiveness Scale (SRS) [49], behavioral and emotional problems will be assessed with the Strengths and Difficulties Questionnaire (SDQ) [50] using both parent and teacher ratings. A developmentally sensitive, general measure of negative self-statements across both internalizing and externalizing problems will be obtained by the use of Children’s Automatic Thoughts Scale (CATS) [51]. In addition, children’s adaptive skills will be assessed with the Adaptive Behavior Assessment System, Second Edition (ABAS-II) [52]. Baseline characteristics regarding socioeconomic status and parenthood will be obtained by questions concerning level of education, employment, household income, etc. In addition, parent’s quality of life will be examined with the WHO-five Well-being Index [53]. Finally, the Experience of Service Questionnaire will assess the children’s and parents’ satisfaction with the treatment [54].

### Sample size

Previous studies have shown that 71.4% of CBT-treated children with ASD and anxiety disorders no longer fulfilled the diagnostic criteria for their primary anxiety disorder (i.e., most severe anxiety disorder based on parents response to ADIS) following treatment, which is markedly greater than for the WL control group, in which none had recovered [25]. Below, we will make the conservative assumption that, at T2, 60% of the CBT participants and 10% of the WL group will no longer fulfil the primary anxiety disorder diagnosis identified at T1 (Fig. 1). Applying a significance level of 5%, it is then estimated that 34 participants (17 in each group) will be needed to obtain a statistical power of 80% to reject the null hypothesis of no difference between these proportions. This calculation is based on a simple two-sided comparison of proportions by Fisher’s exact test without adjustment for blocking, sex, or co-morbidity of disorders other than anxiety disorders. We do not expect the inclusion of these factors to affect the power substantially. Thus, under these assumptions, 50 participants will be enrolled.

### Analysis

The primary outcome will be measured as a dichotomous variable, as one (1) if the primary anxiety disorder identified before treatment (at time point T1) persists post treatment (T2) and as zero (0) if the child no longer fulfils diagnostic criteria for the primary anxiety disorder at T2.

The overall percentage of children free of primary anxiety disorder will be calculated and the effect of the intervention will be measured in terms of odds ratio, comparing odds for recovery in the treatment condition with the corresponding odds in the control condition. This will be calculated by conditional logistic regression and with adjustment for sex and co-morbidity (yes/no) by diagnoses other than anxiety disorders and ASD.

A mixed between–within analysis of variance (ANOVA) design will be used for all continuous outcomes to further evaluate the effect of the CBT intervention. Possible reduction in scale scores on CSR, SCAS, CALIS, ADHD-RS, SDQ, SRS, CATS, and ABAS-II, together with measures of parent’s quality of life, will be investigated to test for differences between CBT and WL groups while comparing pre and post measures within the two groups.

Analysis of the treatment effect such as direct comparison between T1/T1’ and T3/T3’ or changes over time (from T1/T1’ via T2/T2’ to T3/T3’) in the different scale scores may also be considered regarding further elucidation of CBT efficacy.

In order to enhance the strength of the analysis regarding continuation at follow-up (T3 and T3’) of the treatment effect seen at T2 and T2’, the possibility of combining CBT and WL groups will be investigated. However, we are aware that baseline levels at T1’ can be different from T1 due to natural fluctuation, which means that the two samples may not compare well. Thus, combining the samples will only be done after investigating such possible differences at baseline levels.

Repeated measures (longitudinal) analyses will be used when relevant. It is unlikely that the same probability distribution can be used for all secondary outcomes and statistical inference will determine which to apply. For some measures, a normal distribution may be applied, whereas other measures are nominal or at best ordinal.

Though measures will be taken to avoid this, it is expected that a few children in both the CBT group and among the WL will drop-out during the study. The impact of this will be investigated and, if possible, handled by, for example, multiple imputation. Unless participants withdraw the consent to participate, all available data will be used for the analyses in an intention-to-treat analysis under the assumption that data are missing at random. Statistical analyses will be conducted by the use of STATA (StataCorp, College Station, Texas, USA) and the significance level will be set at a *P* value of less than 0.05.

## Discussion

Findings from Australia provide the first evidence for the benefit of this manualized CBT program for children with ASD and co-morbid anxiety disorders [25]. In order to investigate whether the findings regarding the effect of the CBT program can be confirmed in a broader international population this RCT aims to determine the effectiveness of the Cool Kids ASD program in Danish children and, more importantly, aims to demonstrate effects within a real-world, mental health clinic setting.

A limitation of this study could be the choice of anxiety assessment tool since the ADIS-IV-C/P is designed for typically developing children and their manifestations of psychopathology, and might thus not be as reliable and valid in children with ASD [55]. However, no validated anxiety assessment tool for children with ASD is presently available, leaving the ADIS-IV-C/P as the most used assessment tool in this line of research.

Another possible limitation is that a sample of 50 allows for detection of a relatively large effect and, thus, if treatment effects differ by a smaller effect, the study is underpowered. Finally, the use of a WL control condition controls for the simple effects of time, but does not allow conclusions about the specificity of treatment components.

A key strength of this study is its delivery in a real-world clinical setting, using practicing clinical staff. To our knowledge, most similar studies have been placed at university clinics and this will be one of the first studies showing the effect of a manualized CBT program for anxiety in children with ASD in a public mental health clinic setting.

Another strength is the use of outcome measures containing information from a variety of reporters. ADIS-IV-C/P, SCAS, and CALIS are obtained from both parents and children, and ADHD-RS, SDQ, SRS, and ABAS-II from parents and teachers separately. Teachers are found often to be more accurate in evaluating children’s function and behavior, especially with regards to social difficulties, than parents [56].

The literature suggests that structured intervention like the manualized CBT group program will not only improve the main presenting difficulty, but also other aspects of the participant’s functioning such as peer relationships [57]. Training anxiety reduction skills and, thus, decreasing anxiety in children with ASD using the manualized CBT program has the potential of preventing relapse and ensuring better psychosocial development for the child in general. Therefore, the reduction of anxiety symptoms and maybe even of symptoms of other co-morbid disorders may have a great impact on the severity of ASD and, in addition, may aid in improving quality of life for the whole family.

Further perspectives of this study are the contribution to future research in this field and the possibility of translation of an effective treatment for anxiety in children with ASD into daily clinical practice.

### Trial status

A pilot study of five children and their parents was performed in the autumn of 2014, showing almost 50% reduction in the children’s anxiety symptoms (SCAS) [45] and 60% reduction in the interference of anxiety in the children’s lives (CALIS) [46]. In addition, the program received very good satisfaction scores from all the families. A further pilot study with 20 children allocated randomly to CBT or treatment as usual (consisting of five sessions of ASD-based instructions) was performed at the beginning of 2016 (results not yet available). Based on both the pilot studies and the experience from running the program at Macquarie University, Sydney, the treatment manual has been under revision resulting in a second version. This RCT study began in August 2016 and the final group is expected to be recruited and to finish the program by mid-2018.

## Additional file

Additional file 1: SPIRIT (Standard Protocol Items: Recommendations for Interventional Trials). Completed SPIRIT 2013 checklist of recommended items to address in a clinical trial protocol and related documents. (DOC 122 kb)

## Abbreviations

ABAS-II: Adaptive Behavior Assessment System Second Edition
ADHD: attention deficit hyperactive disorder
ADHD-RS: Attention Deficit/Hyperactive Disorder-Rating Scale
ADIS-IV-C/P: Anxiety Disorders Interview Schedule for DSM-IV: Parent & Child interview schedule
ASD: autism spectrum disorder
CALIS: Children’s Anxiety Life Inference Scale
CATS: Children’s Automatic Thoughts Scale
CSR: clinical severity rating
CBT: cognitive behavioral therapy
OCD: obsessive compulsive disorders
SCAS: Spence Children’s Anxiety Scale
SDQ: Strengths and Difficulties Questionnaire
SRS: Social Responsiveness Scale
